# Serum Sulfur-Containing Amino Acids and Risk of Maternal Gestational Diabetes and Adverse Growth Patterns in Offspring

**DOI:** 10.3390/nu15184089

**Published:** 2023-09-21

**Authors:** Ninghua Li, Jing Li, Hui Wang, Yijuan Qiao, Weiqin Li, Ming Gao, Enqing Liu, Zhijie Yu, Gang Hu, Zhongze Fang, Junhong Leng, Xilin Yang

**Affiliations:** 1 Department of Epidemiology and Biostatistics, School of Public Health, Tianjin Medical University, Tianjin 300070, China; m13512929706@163.com (N.L.); leejing1990@hotmail.com (J.L.); wanghuitmu@foxmail.com (H.W.); gaom1997@126.com (M.G.); 2 Tianjin Key Laboratory of Environment, Nutrition and Public Health, Tianjin 300070, China; fangzhongze@tmu.edu.cn; 3 Tianjin Center for International Collaborative Research on Environment, Nutrition and Public Health, Tianjin 300070, China; 4 Project Office, Tianjin Women and Children’s Health Center, Tianjin 300070, China; qiaoyijuan_1984@163.com (Y.Q.); liweiqin007@163.com (W.L.); greaterleq@126.com (E.L.); 5 Population Cancer Research Program and Department of Pediatrics, Dalhousie University, Halifax, NS 15000, Canada; zhijie.m.yu@gmail.com; 6 Chronic Disease Epidemiology Laboratory, Pennington Biomedical Research Center, Baton Rouge, LA 70808, USA; gang.hu@pbrc.edu; 7 Department of Toxicology and Sanitary Chemistry, School of Public Health, Tianjin Medical University, Tianjin 300070, China

**Keywords:** sulfur-containing amino acids, gestational diabetes mellitus, adverse growth patterns, offspring

## Abstract

Background: To estimate associations of sulfur-containing amino acids (SAAs) in the early trimester of pregnancy and gestational diabetes mellitus (GDM) and estimate associations of maternal SAAs with adverse growth patterns in offspring. Methods: We established a 1:1 matched case-control study (n = 486) from our cohort of pregnant women, and 401 children were followed up at ages 1 to 8 years. We conducted binary conditional logistic regression to estimate the risk associations of serum SAAs with GDM. Multinomial logistic regression was implemented to explore associations of maternal SAAs with adverse growth patterns in the offspring. Results: High serum methionine and cystine were independently associated with increased GDM risk (OR: 1.92, 95%CI: 1.18–3.13 and 2.69, 1.59–4.53). Conversely, a low level of serum taurine was independently associated with increased GDM risk (2.61, 1.64–4.16). Maternal high cystine and low taurine were also associated with an increased risk of persistent obesity growth pattern (POGP) in offspring (OR: 2.79, 95%CI: 1.09–7.17 and 3.92, 1.11–13.89) and the effect was largely independent of GDM. Conclusions: High serum methionine, cystine and low serum taurine in the early trimester of pregnancy were associated with a greatly increased risk of GDM. Maternal high cystine and low taurine were associated with elevated risk of offspring POGP, largely independent of GDM.

## 1. Introduction

Gestational diabetes mellitus (GDM) is one of the most common complications of pregnancy and has affected approximately 14.0% of pregnant women globally in 2021, according to an estimate by the International Diabetes Federation [1]. As we all know, GDM might increase maternal risks of diabetes and cardiovascular disease in later life [2,3,4,5] and increase the risk of childhood obesity in offspring [6,7]. However, the effects of management and treatment of GDM are still suboptimal. Lifestyle intervention initiated before the 15th gestational week could only obtain a 22% reduction in maternal GDM risk, but lifestyle intervention of GDM was ineffective in decreasing offspring obesity risk [6,8]. Hence, it is essential to search for novel early biomarkers for maternal GDM and offspring obesity and further explore possible pathophysiological mechanisms for early prediction and better intervention of maternal GDM and offspring obesity.

Sulfur-containing amino acids (SAAs), including methionine, cysteine, cystine and taurine, play major parts in the transmethylation and transsulfuration pathways [9,10]. Dysregulation of these pathways results in changes in methylation patterns on cells and an imbalance in the redox state, which is associated with inflammation and oxidative stress [11,12]. Oxidative stress might impair insulin signaling, contributing to the development of GDM. Increased inflammation can also promote the development of GDM. Recently, several researchers have evaluated the associations between SAAs with insulin resistance and diabetes risk [13,14,15]. Yun et al. found that hepatic methionine and plasma cysteine were markedly lowered in diabetic mice [14]. In a cohort study of obese African-American women matched with BMI and age, plasma cystine was significantly increased in participants with type 2 diabetes (T2D) as compared with those without T2D [15]. However, there are few studies carried out to explore associations between serum SAAs with GDM risk. It is worthwhile to examine whether serum SAAs are associated with GDM risk.

Childhood obesity is one of the concerns in GDM. Undue insulin resistance, hyperglycemia during pregnancy and/or DNA methylation in the fetus are potential risk factors for obesity during childhood. A small study including 64 prepubertal children (5–9 years old) showed that the level of plasma cystine was elevated in healthy obese children while plasma cysteine was not different compared to healthy lean children [11]. The study further found that there were changes in the DNA methylation patterns in obese children, independent of the impaired insulin levels. Prenatal intrauterine environment plays a major part in childhood obesity [16]. Therefore, it is worthwhile, but no research has been implemented to examine risk associations between maternal serum SAAs levels and offspring childhood obesity. However, there are inconsistent findings regarding risk associations between GDM and childhood obesity [17,18]. The group-based trajectory modelling has been employed to classify different patterns of longitudinal BMI changes in childhood [19,20]. It is of interest to explore associations between maternal serum SAAs and adverse growth patterns in offspring and whether GDM mediates the risk associations.

We established a case-control study from our cohort of pregnant women and its follow-up data to examine (1) associations between the serum SAAs concentrations in early trimester of pregnancy and subsequent GDM risk; (2) the associations between maternal SAAs levels in early trimester of pregnancy with obesity-related growth patterns if any, in offspring at 1 to 8 years of age; and (3) whether GDM mediates the risk associations between maternal SAAs concentrations in early trimester of pregnancy and adverse growth patterns in offspring.

## 2. Materials and Methods

### 2.1. Study Design and Participants

The design and methods of this study have been reported previously [21,22]. In short, 22,302 pregnant women were enrolled in the primary hospitals of the Tianjin antenatal care system in 2010–2012. The ethics of this research protocol was approved by the ethics committee of Tianjin Women and Children’s Health Center (TWCHC), and all the participants signed the written informed consent.

At 24–28 weeks of pregnancy, all pregnant women took a 50 g 1 h glucose challenge test (GCT). Thereafter, pregnant women with positive GCT were transferred to the GDM center, where they underwent a standard 75 g 2 h oral glucose tolerance test (OGTT) for diagnosis of GDM. GDM was diagnosed based on the International Association of Diabetes and Pregnancy Study Group’s (IADPSG) criteria [23].

During the fieldwork, 2991 pregnant women out of 22,302 participants were invited and agreed with the provision of overnight fasting blood samples in the first trimester of pregnancy. Among them, we excluded 227 pregnant women who did not have GCT results or whose GCT results were positive but did not have OGTT results. Finally, we included 243 women with GDM and 243 women free of GDM matched by maternal age (±1 year) in the current analysis.

At postpartum, 486 children of the included women were asked to attend the follow-up study and were offered health examinations, including measurement of body height and weight, each year from 1 to 8 years. Finally, 401 children (a response rate of 82.5%) participated in the postnatal follow-up and completed body weight and height measurements at 1 to 8 years of age.

### 2.2. Data Collection

The nurses or obstetricians with uniform training measured maternal weight, height, and systolic/diastolic blood pressure (SBP/DBP) at the first antenatal appointment and offspring height and weight at each follow-up visit aged 1 to 8 years. We also collected other demographic information on maternal age, parity, ethnicity, family history of diabetes, the habits of smoking and drinking, and child gender. We calculate BMI by dividing weight (kg) by squared height (m).

### 2.3. Measurement of Serum Sulfur-Containing Amino Acids

Measurement of serum sulfur-containing amino acids was previously described [24]. Briefly, the blood samples obtained at registration were stored at −80 °C and thawed at 4 °C. After sample pretreatment, it was analyzed by liquid chromatography-tandem mass spectrometry (LC-MS/MS) assay. SAAs in blood samples were identified using PeakView 1.2 and quantified using MultiQuant 2.1.

### 2.4. Statistical Analysis

We performed all statistical analyses using SAS version 9.4 (SAS Institute Inc., Cary, NC, USA). Quantitative data were expressed as mean ± standard deviation (SD) or median (interquartile range) where appropriate. Differences in quantitative data between the two groups were tested using a paired Student *t*-test if normally distributed or a Wilcoxon signed-rank test otherwise. Categorical data were evaluated by the McNemar test or Fisher’s exact test.

Binary conditional logistic regression was conducted to obtain odds ratios (OR) and their 95% confidence intervals (CI) of serum SAAs (methionine, cysteine, cysteine and taurine) for GDM. Restricted cubic spline (RCS) analysis was employed to check the linearity of the associations between SAAs concentrations and GDM risk as before [25,26]. We scrutinized the OR curves of serum SAAs for GDM and identified the optimal cut-off values of serum SAAs at where the risk started to increase rapidly. Then, we classified serum SAAs levels into binary variables at cut-off points. Univariate analysis was used to yield unadjusted ORs and 95%CIs of serum SAAs for GDM risk, and multivariate analysis was conducted to adjust for the traditional GDM risk factors, i.e., pre-pregnancy BMI, SBP, family history of diabetes, smoking habit and weight gain to GCT. At last, we adopted a forward stepwise selection in the conditional logistic regression to obtain serum SAAs that had independent associations with GDM (*p* < 0.05 at entry and exit).

A group-based trajectory modelling method applied to the longitudinal BMI data was performed to identify distinct BMI growth patterns from 1 to 8 years of age [27]. The method offers a data-driven approach to stratify children under study into different developmental trajectory patterns. Using this method, we were able to classify subgroups of children that had different growth patterns, with each subgroup sharing a similar BMI growth trajectory. All parameter estimation and model fitting were performed using a maximum likelihood method. Multinomial logistic regression was employed to yield ORs and 95%CIs of maternal serum SAAs for adverse growth patterns in the offspring, if any. We performed structured adjustment procedures to avoid confounding effects. First, univariate analysis was performed to acquire unadjusted ORs and 95%CIs of SAAs for the risk of adverse growth patterns. Second, multivariate analysis was conducted to adjust for the potential confounding factors, i.e., maternal pre-pregnancy BMI, parity, smoking habit, gestational age at delivery and child gender. Third, we employed the same forward stepwise selection method in the logistic regression to identify independent predictors for adverse growth patterns in the offspring. At last, we adjusted for GDM in the above model to examine the potential mediation effects of GDM between SAAs and adverse growth patterns.

## 3. Results

### 3.1. Baseline Characteristics of Participants

The baseline characteristics of the participating pregnant women are shown in Table 1. Among the participants, the average age is 29.2 (SD 3.1) years, and the average gestational weeks at registration is 10.1 (SD 2.1) weeks. Compared with women free of GDM, those who developed GDM had higher BMI and SBP/DBP. These women were also more prone to be overweight/obese and have a family history of diabetes. The concentration of serum cystine in the early trimester of pregnancy was higher, while serum taurine was lower in GDM women compared with controls. Serum methionine and cysteine were similar between two groups of women.

### 3.2. Associations of Serum SAAs with GDM

**Figure 1:**
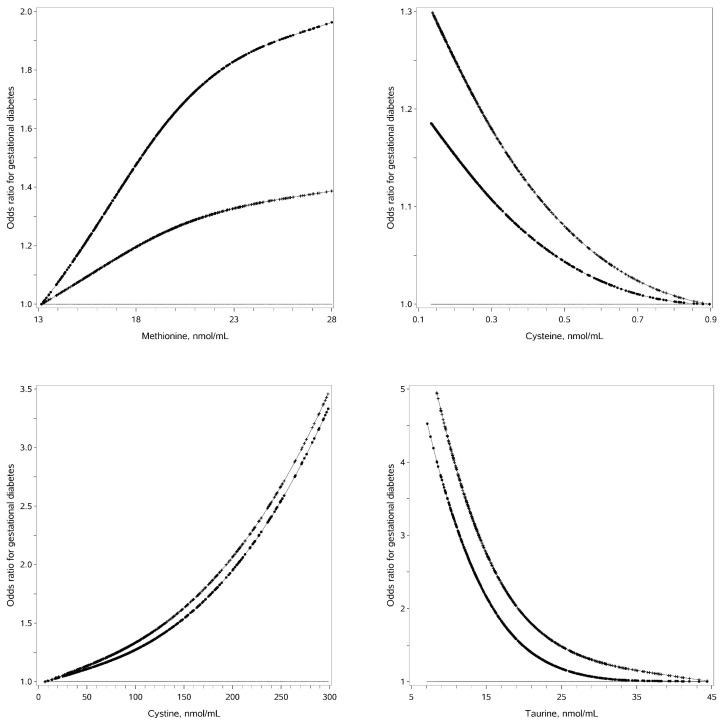
Associations of serum SAAs and gestational diabetes risk. Legends: The straight line is the reference line at odds ratio equal to 1; the dotted line is derived from univariate analysis; the crossed line is derived from multivariate analysis with adjustment for traditional risk factors.

Serum methionine and cystine in the early trimester of pregnancy are positively associated with GDM risk in nonlinear manners, while serum cysteine and taurine are negatively associated with GDM risk in nonlinear manners (Figure 1). Serum methionine ≥ 20.5 nmol/mL, serum cystine ≥ 150 nmol/mL, serum cysteine ≤ 0.38 nmol/mL and serum taurine ≤ 21.9 nmol/mL in early pregnancy are associated with increased GDM risk after adjustment for traditional risk factors (adjusted OR: 1.60, 95%CI: 1.04–2.48; 2.58, 1.58–4.21; 1.52, 1.01–2.27 and 2.14, 1.41–3.26, respectively) (Table 2). The stepwise selection procedure demonstrates that serum methionine ≥ 20.5 nmol/mL, serum cystine ≥ 150 nmol/mL and serum taurine ≤ 21.9 nmol/mL are independently associated with greater GDM risk (OR: 1.92, 95%CI: 1.18–3.13; 2.69 1.59–4.53 and 2.61, 1.64–4.16).

### 3.3. Characteristics of Offspring and Mothers by Different Growth Patterns from 1 to 8 Years of Age

**Figure 2:**
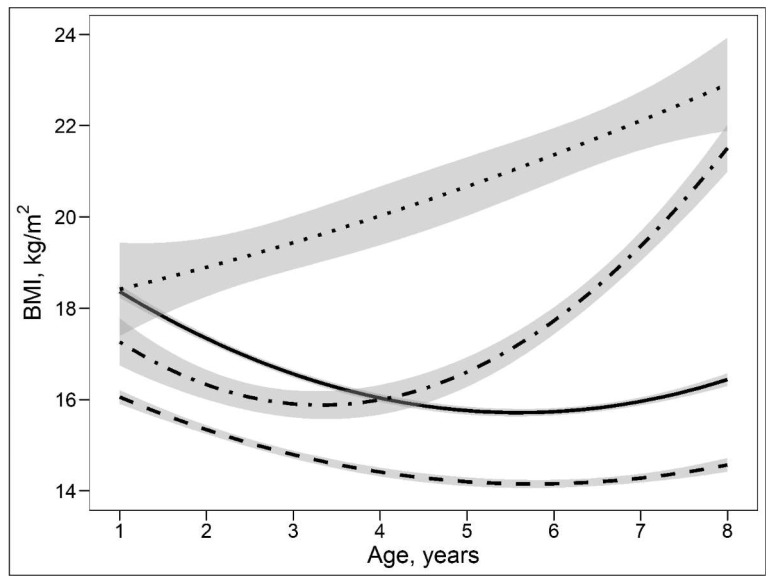
Body mass index trajectories with 95%CIs aged 1 to 8 years based on the group-based trajectory modelling. Legends: the solid line shows a normal growth pattern, the dashed line shows a persistent lean growth pattern, the dotted line shows a persistent obesity growth pattern, and the dot-dashed line shows a late obesity growth pattern.

Based on the group-based trajectory modelling approach, four distinct BMI growth patterns were observed in 401 children who participated in the follow-up visits. The four BMI growth patterns can be described as (1) persistent lean growth pattern (PLGP) characterized by a persistent low BMI over time; (2) normal growth pattern (NGP) characterized by middle and “normal” BMI over time; (3) persistent obesity growth pattern (POGP) characterized by a high and persistent increased BMI over time; and (4) late obesity growth pattern (LOGP) characterized by a normal BMI before 5 years of age and rapidly increased BMI thereafter (Figure 2). Compared with mothers of NGP or PLGP children, their mothers of the POGP and LOGP children have higher BMI, lower concentration of taurine and are more likely to smoke before and during pregnancy (Table 3). Other demographic and clinical characteristics, i.e., maternal age, SBP/DBP, drinking status, gestational weeks at delivery and child gender, were similar among these growth pattern groups.

### 3.4. Associations of Maternal Serum SAAs with POGP and LOGP in Offspring

Maternal cystine ≥ 150 nmol/mL (i.e., high cystine) is associated with markedly increased risk of POGP in the offspring in univariate and multivariate analyses (OR: 3.05, 95%CI: 1.30–7.15 and 2.68, 1.10–6.54). Further adjustment for GDM slightly attenuates the OR of high cystine for POGP, but its statistical significance persists (2.79, 1.09–7.17). However, maternal high cystine is not significantly associated with LOGP risk in offspring. Similarly, maternal taurine ≤ 21.9 nmol/mL (i.e., low taurine) is associated with an elevated risk of POGP, while low taurine is not associated with LOGP risk in offspring (adjusted OR: 3.89, 95%CI: 1.10–13.71 and 2.40, 0.86–6.70). Further adjustment for GDM slightly attenuated the OR of low taurine for POGP, but the statistical significance remained (3.92, 1.11–13.89). On the other hand, maternal methionine and cysteine are not significantly associated with the risks of POGP and LOGP in offspring (Table 4).

## 4. Discussion

Our study explored and confirmed that (1) serum methionine and cystine in early pregnancy were positively associated with GDM risk, while serum taurine was negatively associated with GDM risk in the Chinese population; (2) maternal high serum cystine and low serum taurine in the early trimester of pregnancy were also associated with increased POGP risk in offspring, largely independent of the occurrence of GDM.

In recent years, a few animal and human studies have surveyed the risk associations of SAAs concentrations with insulin resistance and T2D, but their findings were inconsistent. For instance, an animal study found that plasma levels of methionine and cysteine were markedly decreased, and hepatic methionine, cysteine and taurine were also decreased in Zucker diabetic fatty rats [28]. A cohort study of obese African-American women matched with BMI and age reported that plasma cystine was significantly increased in participants with diabetes, and the levels of cysteine and methionine were 21% and 16% elevated, although not significant [15]. Another study included 124 T2D cases and 67 healthy controls also found that plasma cystine was elevated in patients with T2D compared with controls [29]. A small cohort study explored the association between plasma taurine and GDM risk, which found that low levels of taurine were associated with increased GDM risk in multiparous women, but the association was not significant in primiparous women [30]. In our study, we found that high levels of serum methionine, cystine and low levels of serum taurine in the early trimester of pregnancy were independently associated with increased GDM risk.

Biological links between SAAs and GDM remain unclear. SAAs play a major part in the transsulfuration pathway, which can protect against uncontrolled oxidative stress and inflammation through sulfur’s role in redox biochemistry [10,31]. One possible mechanism is that dysregulation of the transsulfuration pathway induces an imbalanced redox state, leading to the occurrence of oxidative stress. Oxidative stress impairs insulin signaling, contributing to the occurrence of GDM. One study assessed the association between dietary SAAs with the status of insulin resistance and oxidative stress and suggested that higher dietary SAAs are associated with adverse metabolic status and the occurrence of oxidative stress [32]. Another possible mechanism is that dysregulation of the transsulfuration pathway results in GDM via inflammation, supported by findings in an animal study that excessive methionine intake could induce a high level of hyperhomocysteinemia and interleukin-6 (IL-6) [33]. An RCT study found that obese women with taurine supplements for eight weeks expressed increased adiponectin and decreased inflammatory biomarkers [34]. Therefore, a low level of taurine might result in elevated inflammation, then promoting the progression of GDM.

In recent years, much research has surveyed the BMI growth patterns in children. A large longitudinal cohort study implemented in the USA identified three distinct BMI growth patterns in children aged 2–6 years using the group-based trajectory modelling method [35]. The three growth patterns were low BMI pattern, median BMI pattern and high BMI pattern, respectively. Using the same method, two other cohort studies conducted in the USA and UK identified four distinct BMI growth patterns in children [36,37]. Both studies identified consistently low growth patterns, late increased growth patterns and consistently high growth patterns. Similarly, using the group-based trajectory modelling method, our study also detected four latent BMI growth patterns in Chinses children from 1 to 8 years of age, i.e., NGP, PLGP, POGP and LOGP.

The associations of maternal SAAs and offspring growth patterns had not been explored. A small research implemented in the USA showed that the concentration of plasma cystine was elevated in healthy obese children while plasma cysteine was not different compared to healthy lean children [11]. The uterine environment has an influence on foetal growth and childhood health. Our study showed that maternal high serum cystine and low serum taurine were significantly associated with increased POGP risk in offspring; the effect was largely independent of the occurrence of GDM.

It is biologically plausible that maternal high serum cystine and low serum taurine were associated with markedly increased POGP risk in offspring. There is growing evidence that epigenetic changes have been a potential mechanistic link between exposure to the uterine environment and childhood obesity in the offspring [38]. The intrauterine environment can impact offspring’s long-term metabolic and obesity risk via epigenetic changes, which has been confirmed in several animal researches relating to maternal nutritional manipulation [39,40,41]. Epigenetic changes refer to heritable alterations in gene expression that do not involve alterations in DNA sequence, of which the most studied mechanism is DNA methylation. SAAs play a major part in the transmethylation pathway, while dysregulation of the transmethylation pathway can result in alterations in the cellular methylation patterns [9,11]. Therefore, it is possible that maternal abnormal SAAs increased childhood obesity risk through epigenetic changes.

GDM can increase the risks of maternal diabetes and cardiovascular disease in later life and childhood obesity risk in offspring [3,5,6], whereas intensive care of GDM was ineffective in decreasing the risks of maternal diabetes and offspring obesity [42,43]. Therefore, it is critically important to identify pregnant women at high risk for GDM at an early stage and identify women whose offspring are at high risk for obesity to design better intervention strategies. In this regard, our study reported that high levels of serum methionine, cystine and low serum taurine in the early trimester of pregnancy were associated with increased GDM risk and further showed that maternal high serum cystine and low serum taurine were also associated with greatly increased POGP risk in offspring. These findings emphasize the importance of maintaining the metabolism normality of SAAs in the prevention of maternal GDM and offspring obesity.

There were some limitations in our study. First, the findings of this study were obtained from a case-control study, which was from a single cohort of pregnant women and their offspring in Tianjin. These findings, therefore, need to be verified in other cohorts. Second, the levels of serum SAAs were related to dietary intake, but we did not obtain detailed diet information from the mothers. Third, homocysteine is an important sulfur-containing amino acid and may play a part in GDM. However, in the targeted LC-MS/MS assay, homocysteine was not specifically included in the measurement scheme. We cannot exclude the possibility that it may have confounded associations between other sulfur-containing amino acids and GDM in this report. Fourth, we noticed that the sample size of the POGP group was small, and the 95%CI of the risk association between maternal low taurine and offspring POGP was large. Further replications by other cohorts are certainly needed to reduce the large type 2 error.

In summary, our study found (1) high concentrations of serum methionine and cystine and low concentrations of serum taurine in the early trimester of pregnancy were independently associated with greatly increased GDM risk, and (2) maternal high concentrations of serum cystine and low concentrations of serum taurine were also associated with greatly elevated POGP risk in offspring, largely independent of GDM. Further studies are warranted to verify our observations, and biological mechanisms underlying the intriguing findings also need exploring for better comprehension of the pathophysiology of GDM and biological links from early life SAAs exposure to adverse childhood growth patterns.

## Figures and Tables

**Table 1:** Baseline characteristics of GDM women and non-GDM women.

Characteristics	Non-GDM (*n* = 243)	GDM (*n* = 243)	*p*
**At registration**			
Gestational weeks	10.1 ± 2.0	10.1 ± 2.1	0.798 *
Age, year	29.2 ± 3.3	29.2 ± 2.7	-
Height, cm	163.2 ± 4.6	163.1 ± 5.0	0.950 *
Weight, kg	58.2 ± 9.6	63.7 ± 10.5	<0.001 *
BMI, kg/m^2^	21.8 ± 3.4	23.9 ± 3.6	<0.001 *
BMI in category			<0.001 **
≥24.0–<28.0	45 (18.5)	77 (31.7)	
≥28.0	12 (4.9)	31 (12.8)	
Systolic blood pressure, mmHg	104.0 ± 10.5	108.3 ± 10.5	<0.001 *
Diastolic blood pressure, mmHg	67.9 ± 7.7	70.6 ± 8.0	<0.001 *
Han nationality	234 (96.3)	238 (98.0)	0.285 **
Education > 12 year	132 (54.3)	135 (55.6)	0.780 **
Parity ≥ 1	12 (4.9)	14 (5.8)	0.683 **
Family history of diabetes	14 (5.8)	30 (12.4)	0.014 **
Smoking before pregnancy	9 (3.7)	10 (4.1)	0.815 **
Drinking before pregnancy	57 (23.5)	72 (29.6)	0.120 **
**Serum sulfur-containing amino acids, nmol/mL**			
Methionine	18.8 (16.4–21.0)	19.3 (16.9–21.8)	0.060 *
Cysteine	0.35 (0.20–0.82)	0.30 (0.21–0.66)	0.359 *
Cystine	91.1 (66.9–129.9)	111.4 (68.8–187.2)	0.001 *
Taurine	21.3 (15.8–27.5)	17.7 (12.8–22.4)	<0.001 *
**At the time of GCT**			
Smoking before and during pregnancy	9 (3.7)	11 (4.5)	0.637 **
Drinking before and during pregnancy	57 (23.5)	73 (30.0)	0.099 **
Weight gain to GCT, kg	8.7 ± 3.2	8.4 ± 3.6	0.128 *

Abbreviations: BMI, body mass index; GCT, glucose challenge test. Data are presented as means ± SD, median (interquartile range), or *n* (%). * Derived from paired *t*-test or Wilcoxon signed-rank test. ** Derived from McNemar test or Firsher’s exact test.

**Table 2:** Odds ratios of sulfur-containing amino acids for GDM risk.

	OR (95%CI)	*p*
**Model 1**		
Methionine, nmol/mL		
<20.5	1.00	
≥20.5	1.71 (1.15–2.55)	0.0086
Cysteine, nmol/mL		
>0.38	1.00	
≤0.38	1.31 (0.91–1.88)	0.1453
Cystine, nmol/mL		
<150	1.00	
≥150	2.50 (1.61–3.88)	<0.0001
Taurine, nmol/mL		
>21.9	1.00	
≤21.9	1.98 (1.35–2.89)	0.0005
**Model 2**		
Methionine, nmol/mL		
<20.5	1.00	
≥20.5	1.60 (1.04–2.48)	0.0341
Cysteine, nmol/mL		
>0.38	1.00	
≤0.38	1.52 (1.01–2.27)	0.0428
Cystine, nmol/mL		
<150	1.00	
≥150	2.58 (1.58–4.21)	0.0001
Taurine, nmol/mL		
>21.9	1.00	
≤21.9	2.14 (1.41–3.26)	0.0004
**Model 3**		
Methionine, nmol/mL		
<20.5	1.00	
≥20.5	1.92 (1.18–3.13)	0.0084
Cystine, nmol/mL		
<150	1.00	
≥150	2.69 (1.59–4.53)	0.0002
Taurine, nmol/mL		
>21.9	1.00	
≤21.9	2.61 (1.64–4.16)	<0.0001

Model 1: univariate analysis. Model 2: multivariate analysis, adjusted for pre-pregnancy BMI, SBP, family history of diabetes, smoking habit and weight gain to GCT. Model 3: forward stepwise regression was employed to obtain SAAs that had independent effects on GDM risk (*p* < 0.05 for entry and exit).

**Table 3:** Maternal characteristics during pregnancy by different offspring growth patterns.

Characteristics	Normal or Persistent Lean Growth Pattern (*n* = 353)	Persistent Obesity Growth Pattern (*n* = 23)	Late Obesity Growth Pattern (*n* = 25)	*p*-Value
**At registration**				
Gestational weeks	10.2 ± 2.0	9.9 ± 2.0	9.4 ± 1.9	0.1725 *
Age, year	29.3 ± 3.0	28.7 ± 2.3	29.8 ± 3.4	0.4406 *
Height, cm	163.3 ± 4.7	162.0 ± 4.9	162.2 ± 5.9	0.2928 *
Weight, kg	60.5 ± 10.4	67.3 ± 11.8	63.8 ± 8.3	0.0040 *
BMI, kg/m^2^	22.7 ± 3.6	25.6 ± 4.3	24.2 ± 2.4	0.0001 *
BMI in category				<0.0001 **
≥24.0–<28.0	87 (24.7)	6 (26.1)	15 (60.0)	
≥28.0	27 (7.7)	8 (34.8)	0 (0.0)	
Systolic blood pressure, mmHg	106.1 ± 10.8	105.7 ± 10.8	106.6 ± 12.4	0.9550 *
Diastolic blood pressure, mmHg	69.0 ± 8.1	70.2 ± 7.6	69.8 ± 8.4	0.7355 *
Han nationality	343 (97.2)	22 (95.7)	24 (96.0)	0.7264 **
Education >12 year	202 (57.2)	10 (43.5)	16 (64.0)	0.3299 **
Parity ≥1	13 (3.7)	0 (0.0)	4 (16.0)	0.0306 **
Family history of diabetes	27 (7.7)	2 (8.7)	4 (16.0)	0.2417 **
Smoking before pregnancy	9 (3.7)	2 (8.7)	2 (8.0)	0.1315 **
Drinking before pregnancy	91 (25.8)	7 (30.4)	6 (24.0)	0.8626 **
**Serum sulfur-containing amino acids, nmol/mL**				
Methionine	19.1 (16.7–21.1)	21.4 (17.6–22.6)	18.5 (16.0–22.0)	0.2373 *
Cysteine	0.31 (0.20–0.77)	0.24 (0.21–0.52)	0.32 (0.22–0.45)	0.7154 *
Cystine	99.0 (69.8–153.9)	151.5 (75.5–221.4)	85.9 (62.0–206.8)	0.2016 *
Taurine	19.4 (13.6–25.5)	17.5 (11.8–20.1)	17.0 (14.1–20.4)	0.0491 *
**During pregnancy**				
Smoking during pregnancy	1 (0.3)	1 (4.4)	0 (0.0)	0.1153 **
Smoking before and during pregnancy	11 (3.1)	3 (13.0)	2 (8.0)	0.0319 **
Drinking during pregnancy	2 (0.6)	0 (0.0)	0 (0.0)	1.0000 **
Drinking before and during pregnancy	91 (25.8)	7 (30.4)	6 (24.0)	0.8626 **
Gestational diabetes mellitus	168 (47.6)	14 (60.9)	18 (72.0)	0.0343 **
Gestational weeks at delivery	39.0 ± 1.7	39.3 ± 1.4	38.6 ± 1.2	0.3441 *

Data are presented as means ± SD, median (interquartile range), or *n* (%). * Derived from ANOVA or Kruskal–Wallis test. ** derived from Chi-square test or Fisher’s exact test.

**Table 4:** Odds ratios of sulfur-containing amino acids for persistent obesity growth pattern and late obesity growth pattern.

	Persistent Obesity Growth Pattern		Late Obesity Growth Pattern	
	OR (95%CI)	*p*	OR (95%CI)	*p*
**Model 1**				
Methionine, nmol/mL				
<20.5	1.00		1.00	
≥20.5	2.20 (0.94–5.14)	0.0682	1.14 (0.49–2.64)	0.7699
Cysteine, nmol/mL				
>0.38	1.00		1.00	
≤0.38	1.47 (0.61–3.55)	0.3947	1.17 (0.51–2.69)	0.7036
Cystine, nmol/mL				
<150	1.00		1.00	
≥150	3.05 (1.30–7.15)	0.0103	1.32 (0.55–3.15)	0.5380
Taurine, nmol/mL				
>21.9	1.00		1.00	
≤21.9	4.23 (1.23–14.50)	0.0218	2.54 (0.93–6.92)	0.0689
**Model 2**				
Methionine, nmol/mL				
<20.5	1.00		1.00	
≥20.5	1.54 (0.63–3.77)	0.3428	1.04 (0.42–2.53)	0.9391
Cysteine, nmol/mL				
>0.38	1.00		1.00	
≤0.38	1.48 (0.59–3.69)	0.4019	1.08 (0.45–2.57)	0.8621
Cystine, nmol/mL				
<150	1.00		1.00	
≥150	2.66 (1.10–6.45)	0.0304	0.96 (0.38–2.43)	0.9277
Taurine, nmol/mL				
>21.9	1.00		1.00	
≤21.9	3.85 (1.10–13.44)	0.0344	2.41 (0.87–6.70)	0.0927
**Model 3**				
Cystine, nmol/mL				
<150	1.00		1.00	
≥150	2.68 (1.10–6.54)	0.0308	0.98 (0.39–2.49)	0.9661
Taurine, nmol/mL				
>21.9	1.00		1.00	
≤21.9	3.89 (1.10–13.71)	0.0345	2.40 (0.86–6.70)	0.0932
Model 4				
Cystine, nmol/mL				
<150	1.00		1.00	
≥150	2.79 (1.09–7.17)	0.0328	0.87 (0.34–2.23)	0.7756
Taurine, nmol/mL				
>21.9	1.00		1.00	
≤21.9	3.92 (1.11–13.89)	0.0340	2.26 (0.81–6.32)	0.1192

Model 1: univariate analysis. Model 2: multivariate analysis, adjusted for maternal pre-pregnancy BMI, parity, current smoker before and during pregnancy, gestational age at delivery and child gender. Model 3: forward stepwise regression was employed to obtain SAAs that had independent effects on adverse growth patterns (*p* < 0.05 at entry and exit). Model 4: further adjusted for GDM on the basis of Model 3.

## Data Availability

The datasets used and/or analyzed during the current study are available from the corresponding author on reasonable request.
